# Supplementary material for: Vitamin D Modified DSS-Induced Colitis in Mice via STING Signaling Pathway
Source: Biology (Basel). 2025 Jun 18;14(6):715. doi: 10.3390/biology14060715 (PMC12190092; doi:10.3390/biology14060715)
Supplement: Supplementary file 1 [file biology-14-00715-s001.zip › Supplementary Materials S1: 16s-report of gut microbiota/Groups/diff_analysis/UnivarTestSpecies/figure/barplot/top20.pdf]

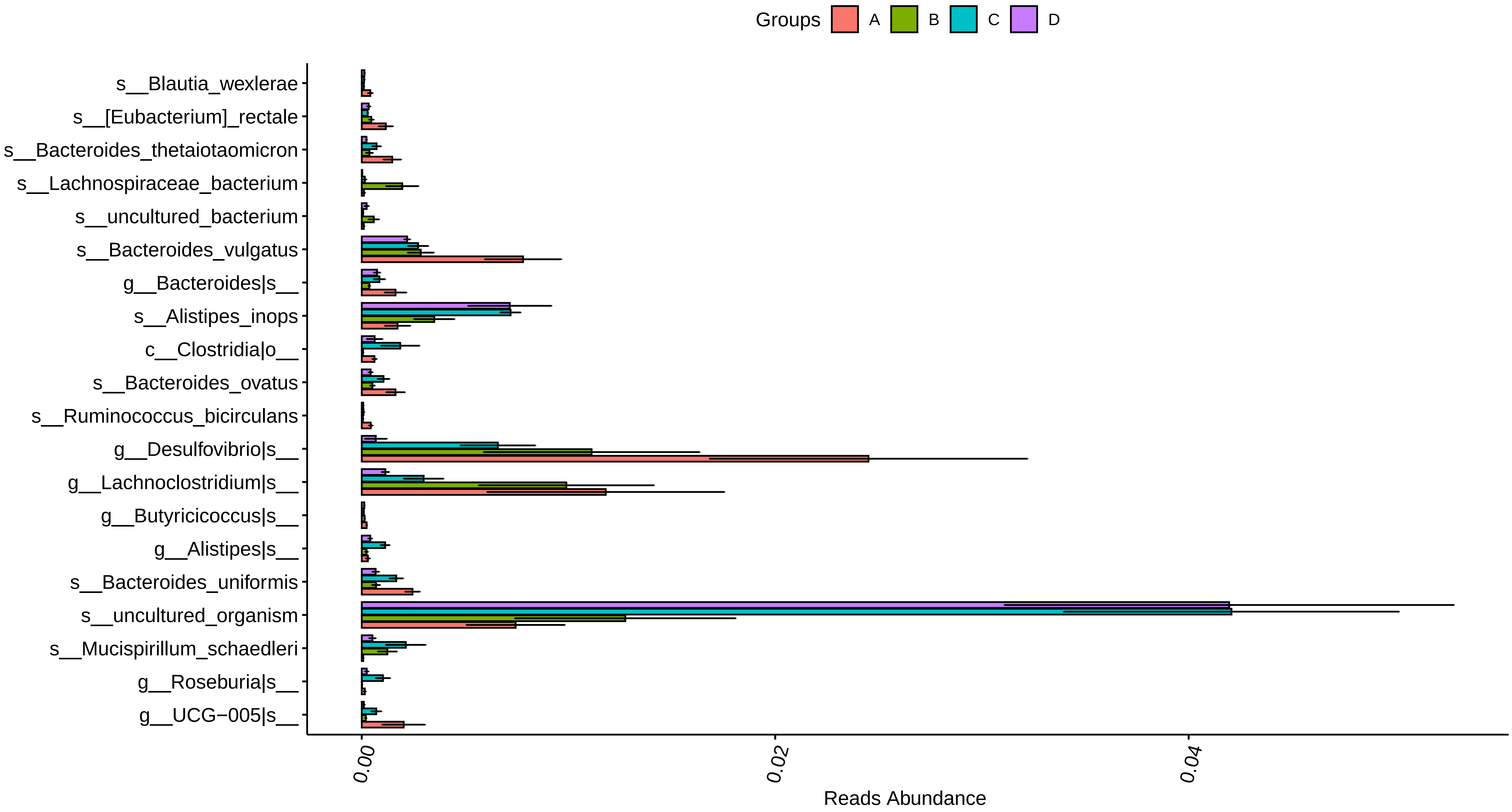

Groups
A
B
C
D
s__Blautia_wexlerae
s__[Eubacterium]_rectale
s__Bacteroides_thetaiotaomicron
s__Lachnospiraceae_bacterium
s__uncultured_bacterium
s__Bacteroides_vulgatus
g__Bacteroides|s__
s__Alistipes_inops
c__Clostridia|o__
s__Bacteroides_ovatus
s__Ruminococcus_bicirculans
g__Desulfovibrio|s__
g__Lachnoclostridium|s__
g__Butyricicoccus|s__
g__Alistipes|s__
s__Bacteroides_uniformis
s__uncultured_organism
s__Mucispirillum_schaedleri
g__Roseburia|s__
g__UCG−005|s__
0.00
0.02
0.04
Reads Abundance
